# Supplementary material for: Prevalence of intestinal parasites in school-age children in Turkey: A systematic review and meta-analysis
Source: PLoS Negl Trop Dis. 2025 Jun 25;19(6):e0013186. doi: 10.1371/journal.pntd.0013186 (PMC12208461; doi:10.1371/journal.pntd.0013186)
Supplement: S1 Table — (DOCX) [file pntd.0013186.s002.docx]

| **Table 1. Database search strategies.** |  |  |
| --- | --- | --- |
| **Database** | **Search strategy** | **Results** |
| Web of Science (all databases) | Search field: (TS=(Parasite OR helmint OR protozoan OR Parasitic)) AND TS=(schoolchildren OR Children OR School); Limits: Restricted to articles, studies in Turkey | 256 |
| PubMed | Search field: ((Parasite[Title/Abstract] OR helmint[Title/Abstract] OR protozoan[Title/Abstract] OR Parasitic[Title/Abstract]) AND (schoolchildren[Title/Abstract] OR Children[Title/Abstract] OR School[Title/Abstract])) AND (Turkey[Title/Abstract] OR Türkiye[Title/Abstract]) | 73 |
| Scopus | Search field: TITLE-ABS ( parasite OR helmint OR protozoan OR parasitic ) AND TITLE-ABS ( child OR school ) AND ( LIMIT-TO ( AFFILCOUNTRY , "Turkey" ) ) | 267 |
| TR İndex | Search field: All fields; Search term: (Parasite OR helmint OR protozoan OR Parasitic) AND (schoolchildren OR Children OR School); No limits used. | 108 |
