# Supplementary material for: Prevalence of intestinal parasites in school-age children in Turkey: A systematic review and meta-analysis
Source: PLoS Negl Trop Dis. 2025 Jun 25;19(6):e0013186. doi: 10.1371/journal.pntd.0013186 (PMC12208461; doi:10.1371/journal.pntd.0013186)
Supplement: S3 Table — (DOCX) [file pntd.0013186.s004.docx]

|  | **S3 Table**. Quality assessment of the included studies | | | | | | | | | | | | |
| --- | --- | --- | --- | --- | --- | --- | --- | --- | --- | --- | --- | --- | --- |
| **No.** | | **Study ID** | **Ref.**  **no** | **Questions assessing included studies** | | | | | | | | | **Yes (%)** |
|  |  |  |  | **1** | **2** | **3** | **4** | **5** | **6** | **7** | **8** | **9** |  |
| 1 | | Aksoy, Ü., et al. (2007) | [13] | Y | Y | N | Y | Y | Y | N | Y | Y | 77.8 |
| 2 | | Tüzemen, NÜ., et al. (2017) | [14] | Y | Y | Y | Y | Y | U | Y | Y | Y | 88.9 |
| 3 | | Ulukanligil, M., et al. (2004) | [15] | Y | Y | U | Y | Y | Y | Y | Y | U | 77.8 |
| 4 | | Vezir, S., at al. (2019) | [16] | Y | Y | U | Y | Y | Y | U | Y | N | 66.7 |
| 5 | | Çeliksöz, A., et al. (2016) | [17] | Y | Y | Y | Y | Y | Y | Y | Y | Y | 100 |
| 6 | | Özkalp, B., et al. (2010) | [18] | Y | Y | Y | N | Y | Y | Y | Y | N | 77.8 |
| 7 | | Yılmaz, EA., et al. (2016) | [19] | Y | Y | Y | U | Y | Y | Y | Y | N | 77.8 |
| 8 | | Simsek, Z., et al. (2004) | [20] | Y | Y | Y | Y | Y | U | U | N | Y | 66.7 |
| 9 | | Yetkin, A., et al. (2010) | [21] | Y | Y | N | Y | Y | Y | Y | Y | N | 77.7 |
| 10 | | Ak, M., et al. (2006) | [22] | Y | N | Y | Y | N | Y | Y | N | Y | 66.7 |
| 11 | | Dagci, H., et al. (2008) | [23] | Y | N | Y | Y | Y | Y | Y | N | U | 66.7 |
| 12 | | Maçin, S., et al. (2017) | [24] | Y | Y | Y | Y | N | Y | Y | Y | Y | 88.9 |
| 13 | | Sankur, F., et al. (2017) | [25] | Y | U | U | Y | Y | Y | Y | Y | U | 66.7 |
| 14 | | Aksoy, Ü., et al. (2003) | [26] | Y | Y | Y | Y | Y | Y | N | Y | N | 77.8 |
| 15 | | Miman, Ö., et al. (2018) | [27] | Y | U | Y | Y | Y | Y | Y | Y | N | 77.8 |
| 16 | | Ekici, A., et al. (2021) | [28] | Y | Y | N | N | Y | Y | Y | Y | U | 66.7 |
| 17 | | Özdil, K., et al. (2020) | [29] | Y | U | N | Y | Y | Y | Y | Y | U | 66.7 |
| 18 | | Bahceciler, NN., et al. (2007) | [30] | Y | Y | N | Y | Y | Y | Y | Y | U | 77.8 |
| 19 | | Tamer, GS., et al. (2007) | [31] | Y | Y | Y | Y | Y | Y | Y | Y | N | 88.9 |
| 20 | | Yilmaz, H., et al. (2008) | [7] | Y | Y | Y | Y | Y | Y | Y | Y | Y | 100.0 |
| 21 | | Artan, MO., et al. (2008) | [32] | Y | Y | Y | Y | Y | Y | Y | Y | N | 88.9 |
| 22 | | Simsek, Z., et al. (2009) | [33] | Y | Y | Y | Y | Y | Y | Y | N | Y | 88.9 |
| 23 | | Çiçek, M., et al. (2012) | [34] | Y | Y | Y | Y | Y | Y | Y | Y | N | 88.9 |
| 24 | | Bacalan, F., et al. (2019) | [35] | Y | Y | Y | Y | Y | Y | Y | Y | N | 88.9 |
| 25 | | Turhanoglu, M., et al. (2012) | [36] | Y | Y | Y | Y | Y | Y | Y | Y | Y | 100.0 |
| 26 | | Eren, C., et al. (2012) | [37] | Y | N | N | Y | Y | Y | Y | Y | U | 66.7 |
| 27 | | Ozdemir, D ., et al. (2005) | [38] | Y | Y | Y | Y | Y | N | Y | Y | U | 88.9 |
| 28 | | Çulha, G., et al. (2006) | [39] | Y | N | N | Y | Y | Y | Y | Y | N | 66.7 |
| 29 | | Aydemir, S., et al. (2024) | [40] | Y | Y | Y | Y | Y | Y | Y | Y | Y | 100.0 |
| 30 | | Aydin, E., et al. (2022) | [41] | Y | Y | Y | Y | Y | Y | Y | N | U | 77.8 |
| 31 | | Türk, S., et al. (2012) | [42] | Y | Y | Y | Y | Y | Y | Y | Y | Y | 100 |
| 32 | | Ciftci, AO., et al. (1999) | [43] | Y | N | U | Y | Y | Y | Y | Y | U | 66.7 |
| 33 | | Girginkardesler, N., et al. (2003) | [44] | Y | Y | Y | Y | Y | Y | Y | Y | Y | 100.0 |
| 34 | | Cemek, F., et al. (2016) | [45] | Y | Y | Y | Y | Y | Y | U | N | N | 66.7 |
| 35 | | Cengiz, ZT., et al. (2015) | [46] | Y | Y | Y | Y | Y | Y | Y | N | U | 66.7 |
| 36 | | Çimen B, and Aktaş O. (2022) | [47] | Y | Y | Y | Y | Y | Y | Y | Y | U | 88.7 |
| 37 | | Güreser, AS., et al. (2022) | [48] | Y | Y | Y | Y | Y | Y | Y | Y | Y | 100.0 |
| 38 | | Çalik, S., et al. (2011) | [49] | Y | Y | Y | Y | Y | Y | Y | Y | U | 88.7 |
| 39 | | Tamer, GS., et al. (2015) | [50] | Y | N | Y | Y | N | Y | Y | Y | Y | 77.8 |
| 40 | | Okyay, P., et al. (2004) | [51] | Y | Y | N | Y | Y | Y | Y | Y | U | 77.8 |
| 41 | | Yentur, DN.,  et al. (2015) | [52] | Y | Y | Y | Y | N | Y | Y | Y | Y | 88.7 |
| 42 | | Doğancı, T., et al. (2002) | [53] | Y | Y | Y | Y | Y | Y | Y | Y | Y | 100.0 |
| 43 | | Ulukanlıgil, M., et al. (2003) | [54] | Y | Y | Y | Y | Y | Y | Y | Y | Y | 100.0 |
| 44 | | Çeliksöz, A., et al. (2005) | [55] | Y | Y | Y | Y | Y | Y | Y | Y | N | 88.7 |
| 45 | | Yentur, DN., et al. (2015) | [56] | Y | Y | Y | Y | Y | Y | Y | Y | N | 66.7 |
| 46 | | Balcıoğlu, IC., et al. (2007) | [57] | Y | Y | Y | N | Y | Y | Y | Y | N | 88.7 |
| 47 | | Babat, SO., et al. (2018) | [58] | Y | Y | Y | Y | Y | Y | Y | Y | Y | 100.0 |
| 48 | | Değerli, S., et al. (2009) | [59] | Y | Y | Y | Y | Y | Y | Y | Y | U | 88.7 |
| 49 | | Ostan, I., et al. (2007) | [60] | Y | Y | Y | Y | Y | Y | Y | Y | U | 77.8 |
| 50 | | Atambay, M., et al. (2007) | [61] | Y | Y | Y | Y | Y | Y | Y | Y | Y | 100.0 |
| 51 | | Turhan, E.,  et al. (2009) | [62] | Y | Y | Y | N | Y | Y | Y | Y | N | 77.8 |
| 52 | | Çamdalı, S., et al. (2024) | [63] | Y | U | N | N | Y | Y | Y | Y | N | 55.6 |
| 53 | | Özkan, AM., et al. (2023) | [64] | Y | Y | Y | Y | Y | U | U | N | Y | 66.7 |
| 54 | | Karakuş, İ., et al. (2022) | [65] | Y | Y | N | Y | Y | Y | Y | Y | N | 77.7 |
| 55 | | Beyhan, Y., et al. (2020) | [66] | Y | N | Y | Y | N | Y | Y | N | Y | 66.7 |
| 56 | | Caner, A., et al. (2020) | [5] | Y | N | Y | Y | Y | Y | Y | N | U | 66.7 |
| 57 | | Taş, ZC., et al.(2019) | [67] | Y | Y | Y | Y | N | Y | Y | Y | Y | 88.9 |
| 58 | | İşler s, et al. (2018) | [68] | Y | Y | Y | Y | Y | Y | Y | Y | U | 88.7 |
| 59 | | Maçin, S., et al. (2016) | [69] | Y | Y | Y | Y | Y | Y | N | Y | N | 77.8 |
| 60 | | Gökşen, B., et al. (2016) | [70] | Y | U | Y | Y | Y | Y | Y | Y | N | 77.8 |
| 61 | | Arıkan, İ., et al. (2016) | [71] | Y | Y | N | N | Y | Y | Y | Y | U | 66.7 |
| 62 | | Yazgan, S., et al. (2015) | [72] | Y | U | N | Y | Y | Y | Y | Y | U | 66.7 |
| 63 | | Tüzemen, NÜ., et al. (2014) | [73] | Y | Y | N | Y | Y | Y | Y | Y | U | 77.7 |
| 64 | | Değerli, S., et al. (2012) | [74] | Y | N | Y | Y | Y | Y | Y | Y | N | 77.8 |
| 65 | | Hamamcı, B., et al. (2011) | [75] | Y | Y | Y | Y | Y | Y | Y | Y | Y | 100.0 |
| 66 | | Ekinci, B., et al. (2011) | [76] | Y | Y | Y | Y | Y | Y | Y | Y | N | 88.9 |
| 67 | | Koruk, I., et al. (2010) | [77] | Y | Y | Y | Y | Y | Y | Y | N | Y | 88.9 |
| 68 | | Güdücüoğlu, H., et al. (2010) | [78] | Y | Y | Y | Y | Y | Y | Y | Y | N | 88.9 |
| 69 | | Köksal, F., et al. (2010) | [79] | Y | Y | Y | Y | Y | Y | Y | Y | N | 88.9 |
| 70 | | Taş, C., et al. (2009) | [3] | Y | Y | Y | Y | Y | Y | Y | Y | Y | 100.0 |
| 71 | | Taş, C., et al. (2009) | [80] | Y | N | N | Y | Y | Y | Y | Y | U | 66.7 |
| 72 | | Ataş, AD., et al. (2008) | [81] | Y | Y | Y | Y | Y | N | Y | Y | U | 88.7 |
| 73 | | Karadam, SY., et al. (2008) | [82] | Y | N | N | Y | Y | Y | Y | Y | N | 66.7 |
| 74 | | Yapici, F., et al. (2008) | [83] | Y | Y | Y | Y | Y | Y | Y | Y | Y | 100.0 |
| 75 | | Malatyalı, E., et al. (2008) | [84] | Y | Y | Y | Y | Y | Y | Y | N | U | 77.8 |
| 76 | | Tamer, GS., et al. (2008) | [85] | Y | Y | Y | Y | Y | Y | Y | Y | Y | 100 |
| 77 | | Yılmaz, H., et al. (2007) | [86] | Y | N | U | Y | Y | Y | Y | Y | U | 66.7 |
| 78 | | Koltas, IS., et al. (2007) | [87] | Y | Y | Y | Y | Y | Y | Y | Y | Y | 100.0 |
| 79 | | Yilmaz, M., et al. (2007) | [88] | Y | 7 | 7 | Y | Y | Y | Y | 7 | 7 | 100 |
| 80 | | Otaǧ, F., et al. (2007) | [89] | Y | Y | U | Y | Y | Y | Y | N | U | 66.7 |
| 81 | | Değerli, S., et al. (2006) | [90] | Y | U | N | Y | Y | Y | Y | Y | U | 66.7 |
| 82 | | Alver, O., et al. (2006) | [91] | Y | Y | Y | Y | Y | Y | Y | Y | Y | 100.0 |
| 83 | | Çelik, T., et al. (2006) | [92] | Y | Y | U | Y | Y | Y | Y | Y | U | 77.8 |
| 84 | | Çeliksöz, A., et al. (2005) | [93] | Y | N | Y | Y | N | Y | Y | Y | Y | 77.8 |
| 85 | | Öztürk, CE.,  et al. (2004) | [94] | Y | Y | N | Y | Y | Y | Y | Y | U | 77.8 |
| 86 | | Coşkun, S. (1991) | [8] | Y | N | Y | Y | N | Y | Y | Y | Y | 77.8 |
| 87 | | Gürses, N., et al. (1991) | [95] | Y | Y | Y | Y | Y | Y | Y | Y | Y | 100.0 |
| 88 | | Balcı, MK., et al. (1990) | [2] | Y | Y | Y | Y | Y | Y | Y | Y | Y | 100.0 |
| 89 | | Öncel, K., (2018) | [96] | Y | N | U | Y | Y | Y | Y | Y | N | 66.7 |
| 90 | | Akış, FB., et al. (2018) | [97] | Y | N | N | Y | Y | Y | Y | Y | N | 66.7 |
| 91 | | Keskinler, D., et al. (1997) | [98] | Y | Y | Y | Y | Y | Y | Y | Y | Y | 100 |
| 92 | | Çelik, T., et al. (2014) | [99] | Y | Y | Y | Y | Y | Y | Y | Y | Y | 100.0 |
| 93 | | Çiçek, M., et al. (2011) | [100] | Y | Y | U | Y | Y | Y | Y | Y | U | 77.8 |
| 94 | | Kaplan, M., et al. (2009) | [101] | Y | Y | U | Y | Y | Y | Y | Y | U | 77.8 |
| 95 | | Börekçi, G., et al. (2009) | [102] | Y | Y | Y | Y | Y | Y | Y | Y | Y | 100.0 |
| 96 | | Malatyalı, E., et al. (2009) | [103] | Y | Y | Y | N | Y | Y | Y | Y | N | 77.8 |
| 97 | | Kaplan, M., et al. (2009) | [104] | Y | Y | N | N | Y | Y | Y | Y | N | 66.7 |
| 98 | | Yaman, O., et al. (2010) | [105] | Y | Y | Y | Y | Y | U | U | N | Y | 66.7 |
| 99 | | Pektaş, B., et al. (2015) | [106] | Y | Y | N | Y | Y | Y | Y | Y | N | 77.7 |

1. Was the sample frame appropriate to address the target population?

2. Were study participants sampled in an appropriate way?

3. Was the sample size adequate?

4. Were the study subjects and the setting described in detail?

5. Was the data analysis conducted with sufficient coverage of the identified sample?

6. Were valid methods used for the identification of the condition?

7. Was the condition measured in a standard, reliable way for all participants?

8. Was there appropriate statistical analysis?

9. Was the response rate adequate, and if not, was the low response rate managed appropriately?

Y=Yes; N=No; U=Unclear or NA Not applicable
