# Supplementary material for: Prevalence of intestinal parasites in school-age children in Turkey: A systematic review and meta-analysis
Source: PLoS Negl Trop Dis. 2025 Jun 25;19(6):e0013186. doi: 10.1371/journal.pntd.0013186 (PMC12208461; doi:10.1371/journal.pntd.0013186)
Supplement: Attachment — Submitted filename: Response to Reviewers.docx [file pntd.0013186.s005.docx]

**Response to Reviewers**

**Original Manuscript ID:** PNTD-D-24-01359

**Original Article Title:** “Prevalence of intestinal parasites in school-age children in Turkey: A systematic review and meta-analysis”

**Dear Editor,**

I express my sincere gratitude to both the editors of the journal and the referees for their insightful reviews and constructive criticisms, which have significantly contributed to enhancing the maturity and quality of our research. I have meticulously addressed each issue raised, responding to the referees' comments in detail.

I am hopeful that our study will be considered for publication in such a prestigious journal.

Best regards,

Asst. Prof Ahmed Galip HALİDİ

**Response to Reviewers**

**Methods**

**Reviewer #1:**

**Concern # 1:** The objectives are articulated (determine pooled prevalence, identify common parasite species, and compare prevalence across regions). However, the hypothesis is not explicitly stated as a testable proposition and could be framed more explicitly.

**Author response:** We sincerely appreciate your constructive feedback, which has significantly contributed to the development of our work. In response to your suggestions, we have incorporated a clear, comprehensible, and testable hypothesis on the subject as requested.

**Concern # 2:** A systematic review and meta-analysis are appropriate for synthesizing prevalence data and comparing regions. The PRISMA guidelines and PROSPERO registration ensure adherence to best practices, yet the PRISMA 2020 statement should be used in place of the outdated 2009 statement.

**Author response:** In accordance with your recommendation, we have revised our study by substituting the PRISMA 2009 diagram with the PRISMA 2020 diagram. I sincerely appreciate your positive and constructive feedback.

**Concern # 3:** While the methodology is sound and robust, a meta-regression addressing the high variability in sample size and study outcomes could shed light on the factors contributing to the observed heterogeneity. This could also allow to test how publication years, diagnostic methods or study settings (urban vs. rural) potentially impact the results.

**Author response:** The meta-regression proposed for this study has been completed, and its findings are thoroughly evaluated in the discussion section. These results are pivotal in underscoring the importance of IPIs within the field of public health. We express our deep gratitude for your significant contribution to the progression of our research.

**Results**

**Reviewer #1:**

**Concern # 4:** The results align with the analysis plan outlined in the Methods section, including the use of random-effects models, subgroup analyses, and tools such as funnel plots for publication bias. However, the results of the quality assessment as well as additional exploratory analyses, such as meta-regression or sensitivity analyses, could help address the heterogeneity observed and could help understand the factors contributing to regional and methodological variability. While the results are clearly presented, the implications of the aforementioned heterogeneity are not fully addressed. Figures and tables are mostly comprehensive, however there are some issues that need to be addressed (please refer to my editorial suggestions).

**Author response:** The issues you identified have been thoroughly examined, and the additional analyses you requested have been conducted, accompanied by the necessary discussions. The discussion section has been expanded to incorporate regional differences. Furthermore, the deficiencies you noted in the editorial suggestions concerning figures and tables have been meticulously addressed. Specifically, the resolution of the forest graphics has been enhanced to a high resolution. We are grateful for your valuable contributions.

**Reviewer #2:**

**Concern # 1:** It is suggested that the images could be improved in resolution. It is very important to see the detail in the forest graphics, but in the current state this is partly compromised.

**Author response:** All forest graphics relevant to the study have been thoroughly reconstructed, with their resolution optimized to the highest setting to ensure detailed visibility.

**Concern # 2:** A better description of the quality of the included studies is needed.

**Author response:** I wish to express my sincere gratitude for your insightful critiques and recommendations, which have substantially contributed to enhancing the efficacy of our study. We have developed a Quality Assessment of the Included Studies table utilizing the Joanna Briggs Institute (JBI) checklist for quality assessment within the manuscript. This table will be included in the appendices under the title "S3 Table Quality Assessment of the Included Studies".

**Conclusions:**

**Reviewer #1:**

**Concern # 5:** The conclusions of this study are well-aligned with the data presented. However, the high heterogeneity across studies raises questions about the reliability of the pooled estimates and regional comparisons. While the manuscript acknowledges heterogeneity, it does not discuss its implications and the authors may consider emphasizing how such variability limits the generalizability and robustness of their conclusions. That said, the authors have identified several important limitations, such as uneven representation of regions and variability in study designs and diagnostic techniques. However, the discussion does not fully address all these points and how these limitations might affect the results.

Overall, the study contributes to a better understanding of intestinal parasite prevalence among school-age children in Turkey, addressing an important area in public health, but the discussion of how these data can inform public health interventions remains limited. Although the authors link the prevalence of intestinal parasites to socioeconomic factors, sanitation, and hygiene, the recommendations remain somewhat broad.

**Author response:** The study's identified deficiencies have been meticulously scrutinized, with particular emphasis on the discussion section, which has been expanded to address these issues. An analysis of how the study's limitations might affect the results has been conducted. Moreover, the suggestions section, which may have initially seemed somewhat general, has been refined to incorporate the socioeconomic, geographical, and other pertinent conditions specific to the country where the research was carried out. We are grateful for your valuable input in enhancing our study.

**Reviewer #2:**

**Concern # 3:** The study is important because it focuses on a fragile public and on very prevalent and neglected diseases. However, there might be room for further exploration in the discussion and a more specific conclusion.

I deeply appreciate the significance of the work, and I believe that a more thorough exploration of the approach could further enhance its impact. Other studies demonstrate the efficacy of state and private interventions on a global scale. It may be beneficial to explore alternative approaches that emphasise addressing challenges and celebrating successes.

**Author response:** Following your insightful recommendations, we have enriched the discussion section by integrating a broader range of literature. Additionally, we have analyzed and contextualized successful practices and suggestions from comparable studies within the scope of our research. I extend my sincere appreciation for your constructive feedback, which has significantly contributed to the enhancement of our study.

**Editorial and Data Presentation Modifications**

**Reviewer #1:**

**Time scope of studies**

**Concern # 6:** Line 88–95: The manuscript mentions the search date (August 2024) but omits details about the time range for the included studies. It is unclear whether there were any restrictions based on publication year. Please specify the publication years of the included studies (e.g., "Articles published between [year] and [year]"). If no time restrictions were applied, please clarify this explicitly.

**Author response:** I extend my sincere appreciation for your constructive feedback. During the course of this study, no temporal restrictions were applied to the database searches and study planning. Articles from all years within the specified databases were evaluated, and those deemed appropriate were included.

**Concern # 7:** Line 155–185 (Characteristics of included studies): Pertaining to the above mentioned point, the omission of the range of years during which the studies were published (or the data of the studies collected) can make it difficult to assess whether the study represents recent trends or includes outdated data. If the prevalence of intestinal parasitic infections is influenced by time-dependent factors (e.g., improvements in sanitation, public health campaigns), it could be interesting to analyze whether prevalence rates have changed over the years.

**Author response:** I express my sincere gratitude for your constructive feedback. In the course of this study, no temporal limitations were imposed during the database searches and study planning. Articles from all years available in the specified databases were assessed, and those deemed suitable were included. All studies up to and including August 2024, the date when the data were reviewed, were considered.

**Data management and study selection**

**Concern # 8:** Line 102–103: The text mentions that "attempts have been made to gain missing data or to clarify any uncertainty with corresponding authors" but does not explicitly state whether any articles were excluded due to non-responses from authors and the exclusion criteria include (in line 112-113) "articles that had limited access and those of authors who did not respond to email two times", implying that such articles were excluded if the authors did not respond after two inquiries.The authors might want to include the number of articles excluded due to non-responses or limited access and discuss whether this had any impact on the results (e.g., potential biases introduced by excluding inaccessible studies).

**Author response:** Given that the number of articles considered for inclusion in the study but subsequently excluded due to non-response or limited access was minimal, and the sample size of the inaccessible studies was also very small, this issue was not addressed in the discussion. It was deemed unlikely to significantly impact the study's results.

**Inclusion and exclusion criteria**

**Concern # 9:** Line 109: The third point in the inclusion criteria ends abruptly with "(3)." without providing the criterion. Please clarify this point.

**Author response:** The phrase "Studies before August 2024" should have been designated as Article 3. This oversight and lack of attention on my part have been rectified following your thorough review and guidance.

**Quality assessment**

**Concern # 10:** The manuscript mentions using the Joanna Briggs Institute (JBI) checklist for quality assessment but does not provide results or explain their impact on the study. A summary table or figure (provided with the supplementary documents) showing the quality assessment results (e.g., high, medium, low) for all included studies (or a subset) could improve transparency, strengthen the manuscript and align with best practices in systematic reviews. Potentially, the authors may consider performing a sensitivity analysis to evaluate how the study quality affects the results. If this is not feasible, discuss the potential influence of study quality in the limitations section.

**Author response:** I would like to extend my heartfelt thanks for your perceptive critiques and suggestions, which have greatly enhanced the effectiveness of our study. In response, we have constructed a Quality Assessment of the Included Studies table, employing the Joanna Briggs Institute (JBI) checklist for quality evaluation within the manuscript. This table is set to be included in the appendices, labeled as "S3 Table Quality Assessment of the Included Studies".

**Limitations**

**Concern # 10:** Line 357–362: The manuscript mentions that only 41 of 81 provinces were covered but does not discuss the potential consequences this could have for the results of their analyses, e.g., the introduction of regional biases or the impact on the generalizability of the findings.

Similarly, while the authors acknowledge that most studies rely on more than one diagnostic method to detect intestinal parasites, no details are provided on the specific methods used across studies or their potential impact, which could be addressed in the limitations section. More information on diagnostic methods could be included in the supplementary materials.

**Author response:** The decision to gather data from 41 out of 81 provinces, acknowledged as a limitation of this study, and its impact on the results have been thoroughly evaluated in the discussion section, with necessary amendments made. In addition, adjustments were undertaken to provide a more comprehensive discussion of the diagnostic methods utilized in the studies incorporated into the research. I extend my heartfelt appreciation for your invaluable contributions.

**Data reporting and accuracy**

**Concern # 11:** Line 159: The reported numbers for studies in the Marmara region appear to be incorrect. Seven studies should have been reported instead of 17.

**Author response:** Upon conducting a meticulous review, it was discovered that the number of studies related to the Marmara region was incorrectly documented as seventeen instead of the accurate count of seven. This discrepancy has been rectified to reflect the correct figure. I extend my sincere appreciation for your thorough and insightful review, which has played a crucial role in enhancing the quality of our article.

**Concern # 12:** Line 214: The authors mention “the free effects model”, which appears to be a typo.

**Author response:** Following your examination, it was identified that the term "free effects model" was erroneously used in place of the correct term "random effects model" in the specified context. The necessary amendments have been implemented to ensure accurate usage. Thank you for your attention to this matter.

**Table and Figure captions**

**Concern # 13:** Line 166–167: Figure 1 has two captions (one within the figure and one below it), which is inconsistent.

**Author response:** The duplication of the label for Figure 1 has been rectified by implementing the necessary amendments.

**Concern # 14:** Line 226–227 (Table 2): While the study explicitly focuses on school-age children (ages 5–18) and all discussions, analyses, and summaries in the text are about school-age children, Table 2, however, references preschool-aged children, which contradicts the rest of the text.

**Author response:** Following your detailed and positive assessment, the error present in the title of the table has been amended.

**Concern # 15:** Line 227 (Table 2): The table includes entries like "Other" without clarifying what "Other" entails, making it less informative.

**Author response:** The term "Other" in Table 2 refers to parasitic agents mentioned in the articles included in the study, which are exceedingly rare in humans and cannot be definitively diagnosed. This categorization was chosen to ensure that no data from our findings were excluded, thereby preserving the research's reliability. We have annotated this with ** as a comment below the table.

**Concern # 16:** Line 230 & 233: Two figures are labeled as Figure 5; the second should be renumbered as Figure 6.

**Author response:** The misnomer has been corrected.

**Language and clarity**

**Concern # 17:** Line 154: "Finally, only 99 (14%) of the articles met the eligibility criteria and included in the systematic review and meta-analysis (Fig 1)." → "Finally, only 99 (14%) of the articles met the eligibility criteria and were included in the systematic review and meta-analysis (Figure 1)."

**Author response:** The necessary correction has been made.

**Concern # 18:** Please revise line 190-192 for clarity and readability: “As a result of the Meta-analysis of the data of the studies included in the study with STATA 18 software, the Funnel plot graph showing the publication bias shows that the publication bias is at an acceptable level.”

**Author response:** The sentence you provided has been re-evaluated, and necessary amendments have been implemented to enhance clarity.

**Concern # 19:** Revise table header names in Table 1 (e.g., "Firs autor. (Year)" → "First Author (Year)" and "Samp Size" → "Sample Size").

**Author response:** I have made all necessary corrections.

**Concern # 20:** Address missing punctuation and tab stops, particularly when listing the intestinal parasites (line 116 - 122), but also in other areas of the manuscript, e.g., in line 22 “204754”, in line 274 “Colombia.[1,9,105–107].”, line 310 “individuals)[17,52]”, line 337 “parasite[14,26,29,36]”, line 374 “Anatolia region”.

**Author response:** I have implemented all the necessary revisions.

**Reviewer #2:**

**Concern # 4:** The study needs a complete textual revision. From grammatical correction to detailed revision of terms such as the names of species that represent serious flaws.

**Author response:** Following the completion of the article's revision, it was meticulously reviewed in collaboration with an expert linguist. Upon identifying the necessary errors, appropriate adjustments were made while adhering to the established terminology. It is my hope that this final edited version of our article will not present significant grammatical challenges.

**Summary and General Comments**

**Reviewer #1:**

**Concern # 21:** The manuscript titled "Prevalence of intestinal parasites in school-age children in Turkey: A systematic review and meta-analysis" addresses a significant public health issue by quantifying the prevalence of intestinal parasitic infections (IPIs) in school-age children across Turkey. Drawing from 99 articles and 204,754 samples, the study reveals regional heterogeneity in prevalence, with a pooled prevalence estimate of 29%, and identifies common parasitic species, and highlights socioeconomic and geographic factors contributing to the burden of disease.

The study aligns well with the journal's scope and offers important contributions to the understanding of IPIs in Turkey, an important public health issue with implications for targeted interventions and policy-making. However, revisions are necessary to improve the manuscript’s clarity, methodological rigor, and presentation.

Its adherence to standard guidelines, large sample size, and identification of regional disparities and common parasites make the study an important and needed contribution to public health research. However, the study is limited by its use of an outdated PRISMA statement, heterogeneity, limited geographic representation, and insufficient exploratory analyses to address the observed variability. The study is methodologically sound and strong, with the large sample size and multi-regional focus adding robustness to the findings, yet the study could benefit from exploring the observed heterogeneity and thus, an enhanced interpretation of the results. A better articulation of the hypothesis and more detailed discussion (e.g., concerning limitations and implications for public health) may also be considered.

**Major revision points:**

While the use of PRISMA 2009 demonstrates a commitment to transparency and methodological rigor, the 2020 update incorporates important improvements and provides a more comprehensive framework. To strengthen the manuscript, I recommend the authors revise their methodology and reporting to align with PRISMA 2020. Further, the authors could consider conducting a meta-regression to identify the factors contributing to the observed heterogeneity, such as sample size, publication year, geographic settings (urban vs. rural), study quality and diagnostic methods. Lastly, I suggest to to enhance the discussion of limitations and their implications, as well as providing region-specific public health recommendations.

**Author response:** As previously mentioned, we have updated our work by replacing the PRISMA 2009 diagram with the PRISMA 2020 version. We are grateful for your constructive feedback, which has played a crucial role in refining our study. In response to your request, we have formulated a clear, comprehensible, and testable hypothesis on the topic. The meta-regression analysis you recommended has been executed, and its results are comprehensively addressed in the discussion section. These findings have significantly highlighted the importance of IPIs in the realm of public health. We appreciate your valuable input in enhancing our research. Additionally, the issues you identified as somewhat broad in the suggestions section have been revisited, with careful consideration of the socioeconomic, geographical, and other pertinent conditions of the country where the study was conducted. We thank you for your contributions to the development of our study.

**Reviewer #2:**

**Concern # 5:** Although the study is nothing new in scientific circles, it represents an important source of information on highly prevalent diseases in a developing country. That alone should be enough to make it relevant and publishable. However, some errors begin to compromise the quality of the proposal presented and the exploration of the data found in the results can go beyond statistical analysis.

**Author response:** We have addressed and corrected all errors identified by the reviewers and our esteemed editors. Additionally, as per your suggestion, the discussion section has been thoroughly expanded and updated to offer a more comprehensive examination of the results.
