# Supplementary material for: Prevalence of intestinal parasites in school-age children in Turkey: A systematic review and meta-analysis
Source: PLoS Negl Trop Dis. 2025 Jun 25;19(6):e0013186. doi: 10.1371/journal.pntd.0013186 (PMC12208461; doi:10.1371/journal.pntd.0013186)
Supplement: Attachment — Submitted filename: Response_to_Reviewers_auresp_2.docx [file pntd.0013186.s006.docx]

**Response to Reviewers**

**Original Manuscript ID:** PNTD-D-24-01359R1

**Original Article Title:** “Prevalence of intestinal parasites in school-age children in Turkey: A systematic review and meta-analysis”

**Dear Editor,**

I would like to express my sincere gratitude to both the editors and the reviewers for their insightful evaluations and constructive criticisms, which contributed significantly to the maturity and quality of our research. It is an honor for me that the changes we have made in the first revision have satisfied the reviewers and you, dear editors. The small parts that you wanted to be corrected have been meticulously handled by us in line with your suggestions and the necessary work has been done.

I hope that our work will be considered for publication in such a prestigious journal.

Best regards,

Asst. Prof Ahmed Galip HALİDİ

**Additional Editor Comments**

**Methods**

**Concern # 1:** Regarding the hypothesis included in the abstract, we have a suggestion for your consideration since the neglected condition is not a hypothesis being tested in the study. Please consider the following suggestion: "The study hypothesizes that the parasite prevalence in school-age children is high, and there is substantial socioeconomic and geographical variation in species-specific prevalence."

**Author response:** We sincerely appreciate your constructive feedback, which contributed significantly to the development of our work. In response to your suggestions, we have included your valuable hypothesis in the abstract.

**Concern # 2:** In the Results section of the abstract, consider changing lines 29/30 to read, "The subgroup analysis revealed that the Southeastern Anatolia is the region with higher prevalence of intestinal parasites among school-age children, with a rate of 41% [...]"

**Author response:** In line with your suggestion, we have changed the wording in line 29/30 as you indicated above. Thank you sincerely for your positive and constructive feedback.

**Concern # 3:** Please correct reference 9. It still refers to the old version of PRISMA (2010)

**Author response:** Reference 9, which I had forgotten to update, has been corrected with the current version. We are deeply grateful for your important contribution to the progress of our research.

**Concern # 4:** Lines 226/228: Consider changing the phrase to: "The funnel plot showed that the publication bias was acceptable."

**Author response:** The statement in line 226/228 has been corrected in line with your suggestions. We express our deep gratitude for your significant contribution to the progression of our research.
